# Housing unit and urbanization estimates for the continental U.S. in consistent tract boundaries, 1940–2019

**DOI:** 10.1038/s41597-022-01184-x

**Published:** 2022-03-11

**Authors:** Scott N. Markley, Steven R. Holloway, Taylor J. Hafley, Mathew E. Hauer

**Affiliations:** 1 Department of Geography, University of Georgia, Athens, USA; 2 Department of Sociology, Florida State University, Tallahassee, USA

**Keywords:** History, Society, Geography

## Abstract

Subcounty housing unit counts are important for studying geo-historical patterns of (sub)urbanization, land-use change, and residential loss and gain. The most commonly used subcounty geographical unit for social research in the United States is the census tract. However, the changing geometries and historically incomplete coverage of tracts present significant obstacles for longitudinal analysis that existing datasets do not sufficiently address. Overcoming these barriers, we provide housing unit estimates in consistent 2010 tract boundaries for every census year from 1940 to 2010 plus 2019 for the entire continental US. Moreover, we develop an “urbanization year” indicator that denotes if and when tracts became “urbanized” during this timeframe. We produce these data by blending existing interpolation techniques with a novel procedure we call “maximum reabsorption.” Conducting out-of-sample validation, we find that our hybrid approach generally produces more reliable estimates than existing alternatives. The final dataset, Historical Housing Unit and Urbanization Database 2010 (HHUUD10), has myriad potential uses for research involving housing, population, and land-use change, as well as (sub)urbanization.

## Abstract (summary)

**Table:** 

Measurement(s)	human dwelling
Technology Type(s)	Geographic Information System
Factor Type(s)	housing units • urbanization
Sample Characteristic - Organism	Homo sapiens
Sample Characteristic - Environment	anthropogenic environment
Sample Characteristic - Location	contiguous United States of America

## Background & Summary

Social and environmental researchers have long aimed to improve how they analyze and understand changes to the built environment. In the United States, investigators frequently rely on multi-decadal, small-area housing unit data from the US Census Bureau to estimate the historical pace and extent of (sub)urbanization, analyze past geographies of housing loss and gain, categorize (sub)urban land types, examine urban morphology, and project future patterns of population growth, development, and land use^1–8^. Such efforts, however, have long been hindered by problems with historical data availability and compatibility. Specifically, the most commonly used small-area census geography, the census tract, did not cover the whole country until 1990 and is redrawn every ten years^9^.

Our dataset fills a distinct niche left by existing data products—notably, HISDAC-US, NHGIS, and LTDB—that attempt to estimate historic subcounty housing units. These datasets, while exceptionally useful and often applied in the broader literature, come with shortcomings that HHUUD10 addresses. For example, HISDAC-US offers historical building counts and floor areas at unrivalled spatial and temporal resolutions^10^. Its source data, however, comes from contemporary property records, subjecting its estimates to substantial survival bias in many cities that underwent “urban renewal” in the mid-to-late twentieth century^11–13^. Additionally, HISDAC-US comes as a raster dataset. Though rasters offer some noteworthy advantages, vector-polygons—namely, census tracts—are much more frequently used in social and demographic research.

The National Historical Geographic Information System (NHGIS) and Longitudinal Tract Database (LTDB) provide some historical housing unit counts in 2010 tract geometries^14,15^. Though shown to produce reasonably dependable population estimates for 2000 data, these data products only go back to 1990 and 1970, respectively. In addition, the LTDB’s pre-1990 data coverage is limited to US cities and metropolitan regions that were tracted in those years^16^. Furthermore, in part because the LTDB focuses on a wide array of variables rather than housing units specifically, its pre-1990 housing data are generated using a coarse interpolation method that is highly susceptible to misallocation^17^. Namely, they rely heavily on error-prone area-weighted interpolation, remove only water surfaces in their dasymetric refinement procedure, and use population weights rather than housing weights to generate their housing unit estimates^14^.

Addressing these gaps in the available datasets, we develop a data product that provides housing unit count estimates in consistent census tract boundaries for every decennial census year from 1940 to 2010 plus 2019 for the entire Continental United States. We mitigate many of the common problems associated with spatiotemporal interpolation—including survivor bias, incomplete coverage, and misallocation—by taking a hybrid approach^18,19^. Combining historical tract records from the NHGIS and land-use polygons from ArcGIS Online, as well as two privately distributed ancillary datasets, we blend and modify a series of well-established spatiotemporal interpolation techniques to generate pre-1990 housing unit estimates in places that contained pre-1990 tracts. These techniques include dasymetric refinement, selective areal weighting, and two variations of target-density weighting^20–22^. For areas that were not tracted in their respective pre-1990 census year, we employ a novel raster overlay procedure that we call “maximum reabsorption.” For 1990 and later, we rely on NHGIS time series data.

We call our data product the **Historical Housing Unit and Urbanization Database 2010 (HHUUD10)**. It consists of an Esri shapefile and GeoJSON file, as well as.csv,.dta,.xpt, and.v8xpt files in long and wide formats. Along with housing unit counts, HHUUD10 includes an estimated “urbanization year” indicating when a given tract surpassed a set urbanization threshold based on its housing density and land cover. The ancillary components used to estimate the urbanization year are also included in the dataset.

Out-of-sample validation reveals that our multi-method, hybrid approach better predicts past housing unit counts in most cases than any one of the component methods alone. We discuss our approach in full in the following section.

## Methods

### Data importation and organization

**Fig. 1:**
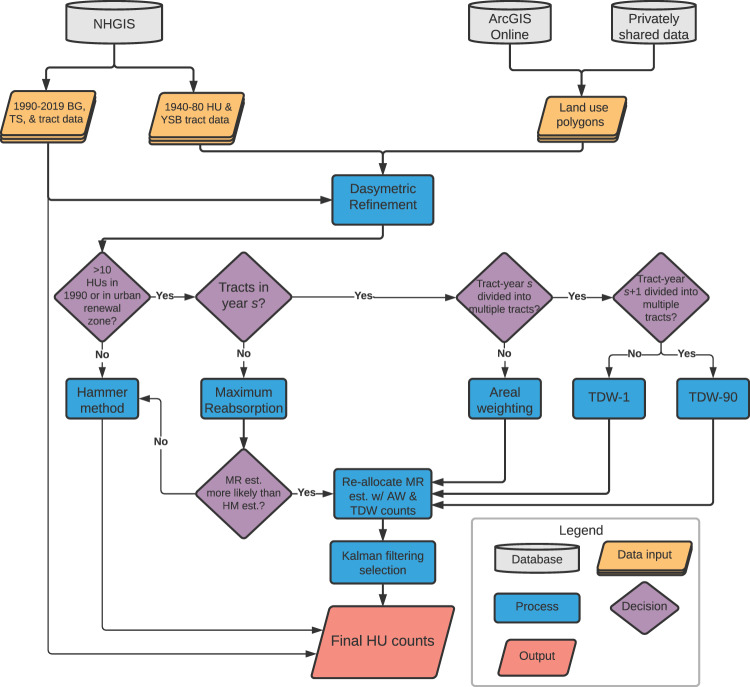
Workflow diagram illustrating the steps we take to produce our housing unit estimates.

In the continental US, there were 72,539 census tracts in 2010. We provide housing unit counts in these tracts across nine decades (1940–2010, 2019), or 652,851 (72,539 × 9) individual tract-years. Fortunately, the NHGIS provides complete estimates for tracts from 1990 and 2000 in 2010 boundaries. Therefore, we generate housing unit estimates for the 362,695 (72,539 × 5) tract-years from 1940 to 1980. We accomplish this by working through the steps outlined in the workflow diagram in Fig. 1. In this subsection, we discuss the data collection and dasymetric refinement procedure.

The data we use to initiate the process come primarily from two sources: the NHGIS and Esri’s ArcGIS Online. From the NHGIS, we first gather historical census data. These include housing units for every census tract in the continental US from 1940 through 1980 plus housing units from the 2010 decennial census and the 2015–2019 American Community Survey (ACS)^15^. In addition, we pull in four other data types from the NHGIS. These include year structure built (YSB) data for 1950-1980 tracts and 1990 block groups, 1990 and 2000 housing unit counts in 2010 tract boundaries from NHGIS’s time series collection (https://www.nhgis.org/documentation/time-series), a crosswalk file that transfers 1990 YSB data into 2010 block groups (https://www.nhgis.org/geographic-crosswalks), and an environmental summary table containing National Land Cover Database (NLCD) land-use categories (https://www.nhgis.org/user-resources/environmental-summaries)^15^. We use this last dataset to generate our “urbanization year” variable, and we supplement it with Enhanced 1992 NLCD data^23^.

From ArcGIS Online, we gather a series of nationwide land-use polygons, which are available to anyone with a standard ArcGIS user license. These layers include water surfaces (https://www.arcgis.com/home/item.html?id=84e780692f644e2d93cefc80ae1eba3a); parks (https://www.arcgis.com/home/item.html?id=578968f975774d3fab79fe56c8c90941); airport grounds (https://www.arcgis.com/home/item.html?id=2706fbe2d7c74b488a609938df8f9578); railyards (https://www.arcgis.com/home/item.html?id=59d26cbc21534cb1b50a37b44d948a53); and golf courses, cemeteries, and industrial areas (https://www.arcgis.com/home/item.html?id=6ffa5cb05c3b4978bd96b8a4b416ffa6). These polygon surfaces indicate which areas likely contained no housing units during our study period. With this information, we are then able to dasymetrically refine our dataset by removing these spaces before interpolation, splitting our pre-1990 tracts into “inhabited” and “uninhabited” zones. Though computationally simple, this binary approach can significantly improve estimates, while often performing as well or better than more complicated techniques^24–26^. Moreover, by using vector-polygons rather than the more standard 30-meter NLCD raster cells we improve the precision of our dasymetric procedure considerably.

One complication of the Esri data is that it does not indicate when the surface polygons were established. However, some of these surfaces have been constructed since 1940, possibly removing housing units in the process. Ideally, we would link each polygon to a construction date. Realistically, this is only feasible for airport grounds and golf courses. For the former, we obtain each airport’s “activation date” from the Federal Aviation Administration (https://adip.faa.gov/agis/public/#/airportSearch/advanced) and then subtract two years to account for construction time. For golf courses, we obtain a georeferenced point file with a year-opened attribute for golf courses in the US that were built by 2000 via private correspondence. These data originate from *Golf Magazine* and have been used in prior research^27^. Dates for both surfaces are then linked to their respective polygons so that dasymetric refinement would only be implemented after their construction. All but two of the remaining polygon surfaces are kept as they were for the entire study period. Swamps are removed from the water file because they can encompass homes, and we only include parks that are less than five square miles because larger parks sometimes do contain residences.

Finally, 2010 tracts containing fewer than 10 housing units in 1990 according to the NHGIS’s time series table are flagged for potential removal. This step corrects for dramatic changes in how the Census Bureau drew tracts during the course of the study period. However, since some of these tracts may have been a site of extreme housing loss due to urban renewal, which we aim to capture, we do not remove any tracts overlapping a known urban renewal project. A polygon boundary of federally funded urban renewal projects carried out between 1950 and 1966 is obtained via correspondence with the Digital Scholarship Lab at the University of Richmond^28^. Housing unit values for these removed tracts are later re-entered using a backcasting procedure discussed below.

**Fig. 2:**
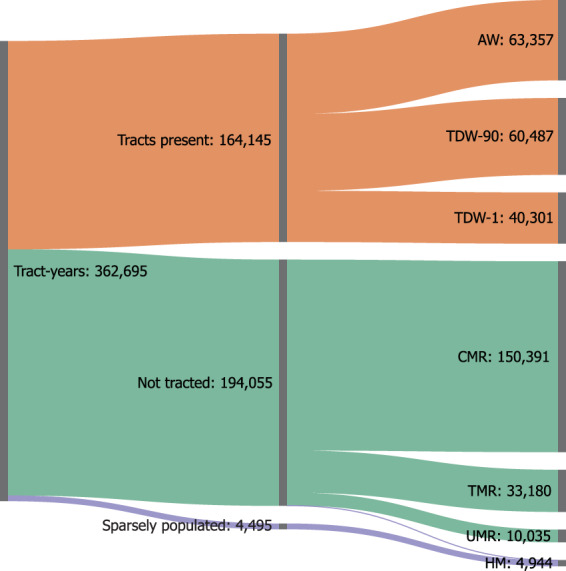
Sankey diagram depicting how our estimation methods are distributed among 1940–1980 tract-years.

Following dasymetric refinement, we sort our 362,695 pre-1990 tract-years into three categories. First, there are target tracts that were covered by tracts in their respective source year (“tracts present”). Next, there are target tracts that were not covered by tracts in their source year (“not tracted”). Finally, there are special-case tracts with less than 10 housing units in 1990 that do not overlap any known urban renewal boundaries (“sparsely populated”). Each of these must be handled in different ways. Figure 2 depicts how tract-years are split into these groups and then subsequently treated with an appropriate interpolation method. We discuss each in the following sections.

### Tracts present

The basic objective of areal interpolation is to reallocate data from one vector-polygon into another. In our case, this entails moving housing unit counts from pre-1990 tracts into 2010 tract geometries. The simplest and most direct method for accomplishing this is areal weighting (AW)^21^. AW is conducted by overlaying source and target tracts, and then using the proportion of the overlapping area to distribute housing units from the former to the latter. The weakness of this approach is that it assumes housing units are distributed uniformly across the source layer, which is rarely the case. Dasymetric refinement is therefore applied to update our assumptions about the within-tract spatial distribution of housing units and improve interpolation results accordingly^20,29^.

Dasymetrically refined AW works well in cases where source geographies remain the same size or are aggregated over time. In such cases, AW can indeed be preferred to other methods because it retains original census housing unit counts and is thus unaffected by survivorship bias. Ideally, we would conduct AW using the smallest census geography, the census block, to minimize misallocation^19^. However, neither census blocks nor block groups are available on a national scale before 1990, and there is not a dependable way to tell if 1990 block data with few or no housing units had housing units in the past. In order to capture housing unit loss, we proceed cautiously and apply dasymetrically refined AW only to tracts that remained the same size or grew over the study period (n = 65,510).

AW works less well in cases where tracts are subdivided between census years. These cases are typically the result of population growth, which rarely occurs evenly within tract boundaries. To address this problem, Schroeder proposes “target density weighting” (TDW)^22^. Like AW, TDW begins with an overlay of the source and target layers. Instead of using the area overlap to proportionally allocate source-year housing units into their target zone (*e.g*., 2010 tracts) though, TDW assumes that the spatial distribution of housing units in the source tract is equivalent to the spatial distribution of a corresponding variable in the target zone. For us, that corresponding variable is the YSB count of housing units built by the source year according to the target-zone data (1990 YSB data reapportioned to 2010 block groups). TDW is thus calculated as

$${\widehat{{\rm{y}}}}_{t}=\sum _{s}\frac{\left(\frac{{A}_{st}}{{A}_{t}}\right){z}_{t}}{{\sum }_{\tau }\left(\frac{{A}_{s\tau }}{{A}_{\tau }}\right){z}_{\tau }}{y}_{s}$$

where, in our case, ${\widehat{{\rm{y}}}}_{t}$ is the estimated housing unit count in target zone *t* (2010 tracts), *y*_*s*_ is the housing unit count in the source year *s*, *z*_*t*_ is the YSB count for source year *s* in target zone *t*, *A*_*t*_ is the area of target zone *t*, *A*_*st*_ is the area of the overlap between source tract *s* and target zone *t* (the “atom”), and *τ* indexes the individual target zones that overlap a given source tract^22,30^.

Ruther and colleagues find that dasymetrically refined TDW outperformed six other interpolation methods when distributing population counts from 1990 and 2000 tracts to 2010 tracts^31^. However, results from 1990 were less reliable than 2000, suggesting that this method is sensitive to temporal distance, especially in places undergoing either population decline or fast growth. TDW is thus ill-equipped to capture the rapid housing unit loss associated with mid-twentieth century urban renewal. However, by already accounting for tracts that stayed the same size or grew in size with AW, our use of TDW is limited to source-year tracts that were subdivided during the study period. Such tracts typically gained population and hence were unlikely to experience dramatic housing loss. And since we use YSB counts from the target zone rather than housing units, the rapid growth problem is effectively neutralized.

Temporal distance remains a concern, however. We address it by applying two different types of TDW that, when combined, minimize temporal distance where possible. For the first, which we call “TDW-1,” we walk housing units from the source year into the tracts in the following decade (*s* + 1)—provided they were subdivided—with TDW using the latter’s YSB counts. Then, for tracts *s* + 1 that stay the same size or grow in area before the target year, we conduct AW. There are 40,416 cases in which TDW-1 is applied, about 72 percent of which are from 1980. Source tracts remaining are those that were subdivided across subsequent decades. For these, we apply a more standard TDW using 1990 YSB counts in 2010 block groups. We call this set “TDW-90,” and we apply it to 59,658 tract-years.

For all three of these methods, we include a quality check step that compares the estimate produced with the tract’s YSB value for the given source year. In theory, the YSB value from 1990 should never be greater than the AW or TDW estimates, except in the relatively infrequent cases wherein a large concentration of older housing units was divided into apartments. To correct these likely mistakes without removing the latter, we keep only the AW and TDW housing estimates that were 90 percent or greater than the YSB count. Cases failing to meet this threshold are handled as if they were non-tracted. Similarly, we keep only the target-zone tracts that were at least 99 percent covered by their source-year tracts, relegating partial overlaps to the non-tracted group.

### Not tracted

**Fig. 3:**
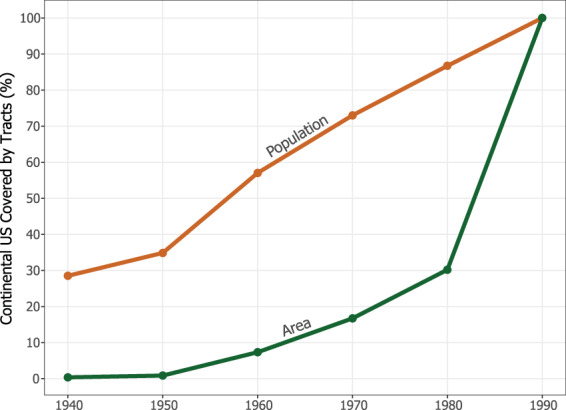
Graph of the percentage of the Continental US’s area and population covered by census tracts, 1940–1990. Since the Census Bureau initially only targeted cities for tract coverage, only a tiny fraction of the total US land area was covered by tracts in the mid-to-late twentieth century, while a considerably greater portion of the total population lived in tracts.

**Fig. 4:**
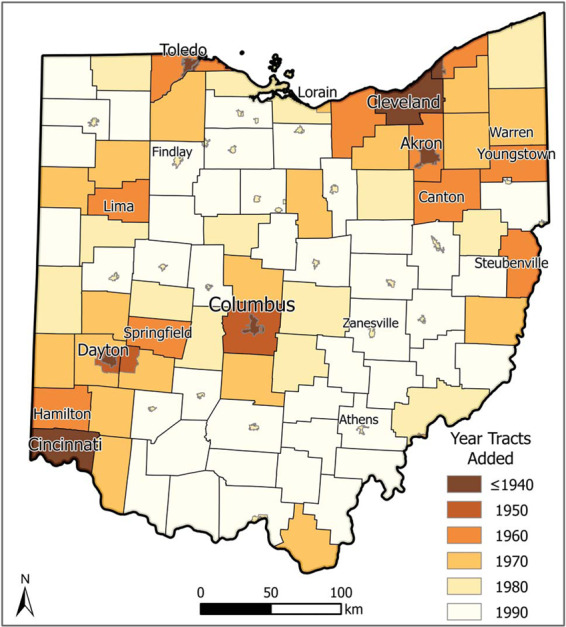
Historical census tract coverage in Ohio, 1940–1990. This map displaying the expansion of tract coverage in Ohio is representative of the general trend across the rest of the country. In 1940, only major cities were tracted. In 1950, tract expansion was limited to a few other cities and areas immediately surrounding a handful of already-tracted places. Over the ensuing decades, tracts were expanded to cover smaller and smaller cities and suburban regions until all areas were tracted in 1990.

In 1940, the Census Bureau had only drawn tracts for a little over 80 cities^9^. In each decade after, coverage was expanded until the entire country was tracted in 1990 (see Figs. 3 and 4). Missing pre-1990 census tract data presents a serious problem for interpolation because the methods described above can only be applied where historical tract boundaries exist. Hammer and colleagues present one solution^4,32^. They use the YSB data in present-day tracts to proportionally allocate county-level housing unit counts available in historical census records. This approach is appealing for its simplicity, and unlike the previous methods discussed, it is not subject to any error from spatial interpolation. However, as with TDW, the Hammer method is sensitive to temporal distance.

A principal assumption guiding us thus far has been that a shorter temporal distance will tend to yield more accurate housing estimates than a longer temporal distance. Therefore, we would prefer to gather housing unit data from pre-1990 tracts where available. However, we also make a countervailing assumption. Over time, the spatial resolution and border accuracy of tracts have generally improved and have grown closer to our target geometries (2010 tracts). Thus, YSB counts in more recent tract-years may provide more accurate housing unit estimates than older tract-years in some places. Our task is then to balance our conflicting preferences for minimal temporal distance and maximal spatial resolution. We thus develop “maximum reabsorption.”

The first step of maximum reabsorption (MR) is to organize YSB totals for the source year (year *s* in Fig. 1) for tracts in every subsequent decade up until 1980, plus in the 2010 block group polygons with 1990 data. If the source year is 1950, for example, the YSB totals for tracts from 1960, 1970, and 1980 and for block groups from 1990 would include their respective YSB counts for “1939 and earlier” summed with their YSB counts for “1940 to 1949.” The resulting total would then indicate the surviving number of housing units built in a given tract by 1950. Once this data is organized for every source year (1940–1980), it is then converted to 30 × 30-meter raster cells. Each raster cell contains an estimated YSB count, calculated as its tract’s YSB count divided by the number of cells contained within that tract. YSB counts are hence assumed to be evenly distributed within each dasymetrically refined tract polygon.

Following rasterization, each source year is left with a set number of associated raster layers, each with their own YSB-derived housing unit estimates. There are six rasters for 1940 (1940–1990), five for 1950 (1950–1990), and so on. Here, we arrive at that crucial juncture: how do we weigh temporal distance against spatial resolution? Our solution is to overlay each raster layer for each source year and then extract the *maximum* housing unit count by raster cell. The logic is the same as before: YSB counts for a given source year in a future tract-year should theoretically never be greater than the original housing unit counts, except where there is a high concentration of older homes that have been divided into multiple units or where formerly non-residential buildings are converted into apartments or condominiums. However, since these practices have historically been observed in urban centers, these cases have mostly been handled by AW or TDW^33,34^. Therefore, the only reason a more recent YSB count should be greater than an older YSB count in remaining raster cells is if the more recent census geometry covers a smaller area than the older tract. Using the maximum thus favors spatial resolution over temporal distance in these cases and the reverse in all other cases.

The next step in the process is to “reabsorb” the new “maximum” cells back into target tracts. This is conducted in ArcGIS Pro using zonal statistics to sum each cell value in its 2010 tract boundary, producing a housing estimate for each source year in their respective target tract. These figures are not final, however. Using maximums implies that housing counts are overestimated in some places. To mitigate this potential issue, we use our MR estimates as weights to proportionally distribute historical county-level housing unit counts into 2010 tract boundaries. Counties that changed size or shape during the study period (*e.g*., many of Virginia’s county-equivalent cities) are amassed into larger county-units to ensure proper allocation.

We call this first cut “County-based Maximum Reabsorption,” or “CMR,” However, there are two potential adjustments to be made. First, there are cases in which the proportional allocation procedure reduces the CMR value below 90 percent of its YSB value. In these cases, the unadjusted maximum was likely the more dependable value, so we either kept it unadjusted (UMR) or used the Hammer method (HM) instead. We choose the value that is closer to the 1990 YSB count because this generally yields the more conservative estimate. In 9,859 cases, this leaves us with the UMR count, and in only 439 cases, it gives us the HM count. After subbing in these new values, CMR estimates for the remaining tracts are recalibrated.

The second adjustment looks to improve our MR estimates with “Tract-based Maximum Reabsorption,” or “TMR.” By subtracting AW and TDW estimates from county totals, we can reduce the number of MR-estimated tract-years in need of reallocation. This should, in theory, improve our maximum reabsorption figures in all county-years with partial tract coverage. Unfortunately, census data itself is not always consistent, especially in older census years (https://www.nhgis.org/frequently-asked-questions-faq#1960_Data)^15^. For example, since tracts are nested within counties, county housing counts should equal the sum of their tract counts. However, there are some significant discrepancies. In the worst of these cases—Kings County, NY (Brooklyn) in 1960—there are over 35,000 more housing units in the county-level census file than in the sum of that county’s census tracts.

Unless TMR comes back as a negative value, there are few ways to identify which method yields the more dependable result. To help us in this task, we employ Kalman filtering for tracts with at least one source year relying on maximum reabsorption. Kalman filtering is a univariate time series approach that forecasts—or, in our case, backcasts—estimates using existing observations^35^. Thus, we use the 2019, 2010, 2000, and 1990 housing unit counts already in our dataset, in addition to any other produced by AW, TDW, UMR, or HM, to generate a predicted housing count for a given tract-year. Then, we select the CMR or TMR estimate that is closer to the Kalman estimate. Since this approach requires observed data on both ends of the projection to work properly, we impute missing 1940 values by selecting either the CMR or TMR estimates that is closer to the HM estimate. These procedures yield 149,231 CMR estimates and 33,087 TMR estimates.

### Sparsely populated

The final tract-years in need of estimation represent only slightly more than one percent of the total (n = 4,495). These special cases are primarily constituted by large sparsely populated zones—including airports, parks, commercial or industrial districts, federal lands, and the like—that were assigned their own tract by the Census Bureau in 2010. Assigning these sparsely populated zones to their own tract, rather than subsuming them in their neighbor’s populated tract, is a relatively recent practice. Thus, when redistributing data from old census tracts to recent ones, there is a high risk of misallocation in these instances if not appropriately managed.

We specified how we identified these cases above. These are tracts with less than 10 housing units in 1990 that did not overlap a known urban renewal area. We handle these rare cases by removing them from the dataset before interpolation before reintroducing them after. We then perform the Hammer method using their YSB estimates and the YSB estimates from all other tracts in their county equivalent.

### Urbanization estimation

Our final task is to provide urbanization year estimates in each 2010 tract. We do this in two cuts. First, following the common practices of housing researchers, we define a tract as “(sub)urbanized” when it surpasses 200 housing units per square mile^36–38^. Notably, the area used for calculation is a tract’s populated dasymetric zone not including its “industrial areas.” We call this “UY1” (Urbanization Year One), and it spans from 1940 to 2019. Once a tract surpasses this threshold, our definition does not allow it to become “unurbanized,” though HHUUD10 users can easily change this feature if they wish.

**Fig. 5:**
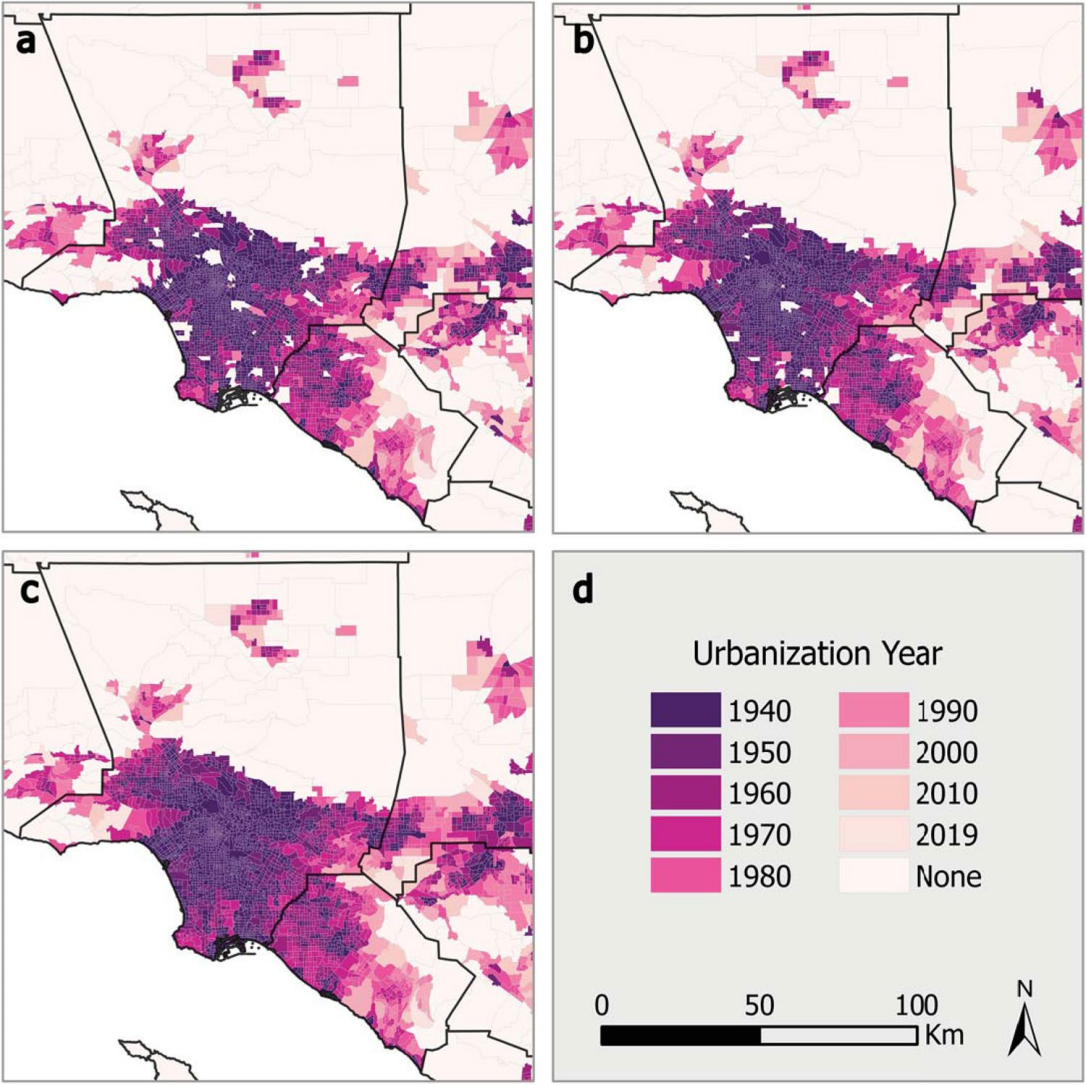
Maps comparing three ways to define urbanization years (UY) in Greater Los Angeles. All use 200 housing units per square mile. (**a**) Hammer-generated UY. (**b**) HHUUD10-generated UY using housing units only (UY1). (**c**) HHUUD10-generated UY supplemented with NLCD and tract adjacency information to account for non-residential urbanized tracts (UY2). (**d**) Map elements.

UY1 captures most tracts one would expect. However, it still leaves conspicuous holes on the urban landscape (see Fig. 5). There are many places typically considered “urban” that do not contain many housing units and have not been removed during dasymetric refinement, such as large commercial and industrial districts. To account for these other urban land uses, we pull in NLCD categories from 1992, 2001, and 2011. Conveniently, the NHGIS has distributed 2001 and 2011 NLCD land cover types in 2010 tract boundaries. To estimate the percentage of land area covered by an urban land use, we simply sum the four “Developed” land cover classifications and then divide them by the total land area minus “Open Water” and “Perennial Ice/Snow.” This produces a “percent developed” category we use to update UY1 into UY2.

NLCD data from 1992 is not as conveniently packaged. It is only available in its original raster format. For that data, we replicate the methods of the NHGIS to aggregate NLCD classifications into our target tracts and then perform the same tasks outlined above. The major distinction is that the NLCD calculated and named the 1992 classifications slightly differently. Rather than using “developed” classifications, we approximate urban land uses by summing “Low” and “High Intensity Residential,” “Commercial/Industrial/Transportation,” “NLCD/LULC Forested Residential,” and “Urban/Recreational Grasses”^23^.

The output from these procedures are three data columns in our dataset indicating the percentage of land that was developed in 1992, 2001, and 2011. With that information, we identify tracts for each NLCD year with an urban land coverage above 50 percent and an urbanization year later than 1990, 2000, and 2010, respectively. For these special cases, we calculate a UY2 value using the weighted average of their neighbors’ UY1 values. The weights in this equation are their shared border lengths. For purposes of averaging, tracts that were not yet urbanized are given a pseudo UY1 of 2035.

**Fig. 6:**
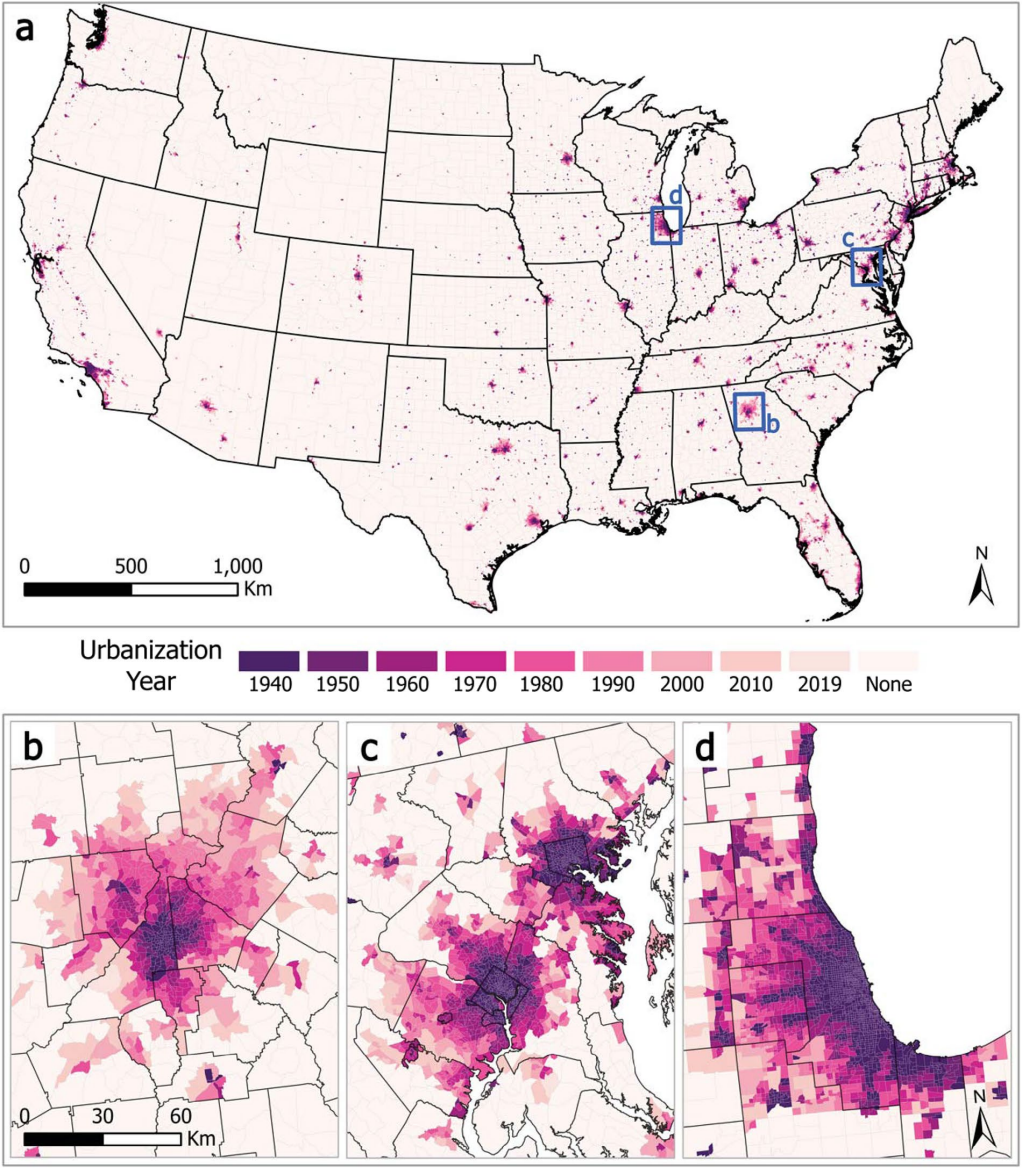
HHUUD10 Urbanization Years by tract for (**a**) the Continental US, (**b**) Greater Atlanta, GA, (**c**) the Baltimore-Washington, DC-MD-VA Area, and (**d**) Greater Chicago, IL-IN-WI.

Following these modifications, we conduct an urban smoothing technique that assigns tracts surrounded by neighbors with earlier urbanization years a new UY2 value using the latest bordering UY2 value. For example, a 2000 UY2 tract surrounded by 1970 and 1980 UY2 tracts would receive a new UY2 value of 1980. The theoretical basis here is that these islands are less likely to be categorically distinct from their neighbors than to be the result of the inherent limitations of using vector polygons to capture urban development. Following this step, we have the final data product, presented in map form in Fig. 6. It includes housing unit counts and the “inhabited” surface areas from 1940 to 2019; a “percent developed” category for 1992, 2001, and 2011; and two urbanization years, one solely housing density based (UY1), the other corrected to include non-housing unit urban land uses (UY2).

## Data Records

All HHUUD10 data are available for download at the Open Science Foundation (OSF)^39^. This repository includes an Esri shapefile and GeoJSON file, as well as.csv,.dta,.xpt, and.v8xpt files in long and wide formats. The data contained in each file are identical. Table 1 defines and summarizes the data columns in the long HHUUD10 file.

**Table 1:** Data description for HHUUD10.

Variable	Description
STATE	State abbreviation.
COUNTY	County name.
GISJOIN10	Unique tract ID in NHGIS format.
GEOID10	Unique tract ID in Census Bureau format.
YEAR	Year of HU & SQMI data (1940-2019). In wide format, the last two digits in the year trail the variable name (e.g., hu40, sqmi40, etc.).
HU	Tract housing unit estimate by year.
SQMI	Area of dasymetrically refined tract in sq. mi. by year. These values are constant throughout the study period except are reduced when an airport or golf course is constructed.
pdev92	Percent of a tract’s land area that was covered by an urban land use according to the NLCDe 1992.
pdev01	Percent of a tract’s land area that was covered by an urban land use according to the NLCD 2001.
pdev11	Percent of a tract’s land area that was covered by an urban land use according to the NLCD 2011.
UY1	Urbanization year according to when a tract surpassed 200 HU / sq. mi. in its dasymetrically refined area.
UY2	Same as UY1, except urbanized non-residential areas identified by the pdev variables and tract adjacency are included.

## Technical Validation

To evaluate how our HHUUD10 method performs against other methods, we apply three validation tests. The first two compare results from our hybrid method against those from its component methods. The third shows the distribution of the estimated error in tracted and non-tracted spaces in our validation sample. To conduct these tests, we use nine sample counties to generate 1990 housing unit estimates in 2010 tract geometries with NHGIS time series data as the “observed” data. Although this dataset itself is imperfect, it is the industry standard and has been shown to outperform alternative datasets^16^.

Other than the historical availability of tract data, the main factor that can skew our estimates is the rate at which an area gains or loses housing units between the source and target years. Thus, following previous studies, we run our validation on a sample of counties with different growth trajectories from 1990 to 2015–19^30,31^. We include three counties that experienced housing unit decline in that time, three that experienced rapid growth, and three that were relatively stable. Table 2 shows the breakdown.

**Table 2:** Nine county sample used for validation.

Growth Trajectory	County	Primary City	Housing Units (1990)	Housing Units (2015-19)	Change
Declining	Orleans Par., LA	New Orleans	225,573	191,808	−33,765
	St. Louis city, MO	St. Louis	194,919	176,729	−18,190
	Wayne Co., MI	Detroit	832,710	815,102	−17,608
Growing	Orange Co., FL	Orlando	282,686	535,981	253,295
	Tarrant Co., TX	Fort Worth	491,152	767,808	276,656
	Riverside Co., CA	Riverside	483,847	840,501	356,654
Stable	Allegheny Co., PA	Pittsburgh	580,738	600,399	19,661
	Essex Co., NJ	Newark	298,710	317,314	18,604
	Hamilton Co., OH	Cincinnati	361,421	379,402	17,981

For our first validation test, we compare our hybrid model of dasymetrically refined (DR) AW, TDW-1, and TDW-90 against DR-AW and DR-TDW alone. Results are presented in Table 3. We use two statistics of comparison: Median Absolute Percent Error (MdAPE) and symmetric Mean Absolute Percent Error (sMAPE). Unlike Mean Absolute Percent Error (MAPE), these measures avoid having to divide by zero and are more resilient against extreme values^40^. In all growth scenarios, as well as among the total, our HHUUD10 method performs better by these measures than both DR-AW and DR-TDW alone. The differences between HHUUD10 and DR-TDW are quite close, however. One reason this may be is that the NHGIS relies heavily on DR-TDW to produce their estimates—though they do this at the block level, use housing counts instead of YSB, and employ a different DR process—so there is some risk of tautology^15^. While DR-AW performs nearly on par with HHUUD10 and DR-TDW in declining and stable counties, its large errors in growing counties demonstrate why AW should only be used in specific cases.

**Table 3:** Comparing 1990 housing unit estimates in 2010 tract geometries: HHUUD10 vs. DR-AW and DR-TDW using Median Absolute Percent Error (MdAPE) and symmetric Mean Absolute Percent Error (sAPE).

County Type	Method	MdAPE	sMAPE	tracts (n)
Declining	HHUUD10	*0.003*	*0.027*	891
	DR-AW	0.004	0.053	
	DR-TDW	0.003	0.032	
Growing	HHUUD10	*0.061*	*0.228*	1017
	DR-AW	0.241	0.452	
	DR-TDW	0.137	0.293	
Stable	HHUUD10	*0.013*	*0.056*	834
	DR-AW	0.016	0.073	
	DR-TDW	0.014	0.056	
Total	HHUUD10	*0.012*	*0.111*	2742
	DR-AW	0.018	0.207	
	DR-TDW	0.016	0.136	

Italicized figures indicate the lowest errors in their group.

For our second validation test, we mimic historical census tract availability by removing two-thirds of 1990 tracts and one-third of 2000 tracts. Following historical patterns, we include only those tracts around the urban center in 1990 and then radiate outward from there. Though imperfect, this scenario allows us to compare HHUUD10 against CMR and HM estimates. Additionally, it allows us to see how substituting HM estimates in for CMR estimates would change our HHUUD10 counts. Table 4 reports the results.

**Table 4:** Comparing 1990 housing unit estimates in 2010 tract geometries: HHUUD10 vs. HHUUD-HM, CMR, and HM using Median Absolute Percent Error (MdAPE) and symmetric Mean Absolute Percent Error (sAPE).

County Type	Method	MdAPE	sMAPE	Tracts (n)
Declining	HHUUD10	*0.046*	*0.123*	891
	HHUUD-HM	0.074	0.151	
	CMR	0.068	0.140	
	HM	0.136	0.211	
Growing	HHUUD10	*0.106*	0.211	1017
	HHUUD-HM	0.110	0.217	
	CMR	0.117	0.229	
	HM	0.114	*0.206*	
Stable	HHUUD10	*0.050*	*0.093*	834
	HHUUD-HM	0.058	0.114	
	CMR	0.072	0.113	
	HM	0.080	0.148	
Total	HHUUD10	*0.066*	*0.146*	2742
	HHUUD-HM	0.083	0.164	
	CMR	0.083	0.165	
	HM	0.106	0.190	

Italicized figures indicate the lowest errors in their group.

Errors in this test are considerably higher than their counterparts in the previous test. This reflects what happens to the accuracy of estimates when historical tract data is unavailable. Still, the HHUUD10 method outperforms the other methods by both measures among declining and stable counties and overall. In fast growing counties, HHUUD10 has the lowest MdAPE, but the Hammer method has a slightly lower sMAPE. This is likely due to the fact that the Hammer method is not subject to any error associated with areal interpolation, and although the HM is affected by survivorship bias, that is much less of a concern in fast-growing places in this time period. Applying the HM over a longer time period when housing demolition was more rampant, such as during the height of urban renewal in the 1950s and 1960s, would assuredly yield less reliable estimates.

**Fig. 7:**
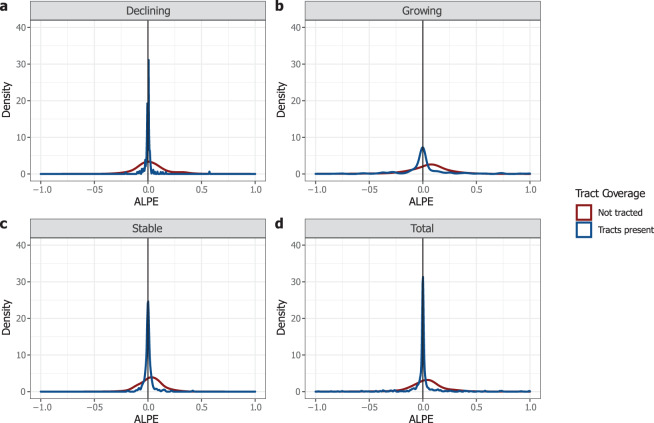
Algebraic Percent Errors (ALPE) in tracted and non-tracted spaces. (**a**) ALPE in Declining counties (**b**) ALPE in Growing counties (**c**) ALPE in Stable counties (**d**) ALPE in all nine sample counties.

Our third test separates tracted from non-tracted spaces to demonstrate the distribution of their errors around zero (see Fig. 7). The error metric used here is the Algebraic Percent Error (ALPE), which is the same as sMAPE shown above, except it does not take the absolute value^41^. The density plots in Fig. 7 show that, as we would expect, the error for tracted spaces tends to be much smaller on average than the error in non-tracted spaces. This is especially true in declining counties where the areal interpolation methods are best at predicting housing counts. The distribution of errors is much wider in non-tracted spaces for all growth trajectories, suggesting that HHUUD10’s estimates are likely less dependable, on average, in earlier census years and in less populated counties where tract coverage was less complete.

## Usage Notes

HHUUD10 provides housing unit counts and urbanization estimates for every census tract in the continental US in consistent boundaries. The data itself may be used for a longitudinal study directly addressing questions about housing, (sub)urbanization, or land-use change, or it may provide a useful input or control variable for studies focusing on other pertinent questions. This data may also help researchers reallocate other census variables into consistent tract boundaries. For any of these potential uses, users should keep three limitations in mind.

First, users of HHUUD10 should note that our dataset includes the sum total of housing units in a given tract-year. Thus, HHUUD10 does not allow for an exact assessment of how many housing units were demolished or constructed. For example, if ten housing units were demolished and subsequently replaced with twenty housing units within a census decade, HHUUD10 only registers the additional ten units. It does not register ten units lost and twenty gained. Researchers using HHUUD10 should design their studies with this in mind.

Second, we recommend that users acknowledge the intention and limitations of our “urbanization year” calculation. This estimate is designed to be used primarily as an input variable in studies involving (sub)urbanization, land-use change, and the like. It hence reflects a specific, density- and land-use-based definition of “urbanization” that does not allow tracts to ever become “unurbanized.” Researchers aiming to capture “de-urbanization,” for example, can use the other variables in the dataset to construct an alternative urbanization definition that is more suitable for their needs.

Finally, at every step in our interpolation procedures, we choose the method previous studies and our own expertise suggest will produce the most accurate possible estimate. Still, no data interpolation process is without error, and as our validation tests show, this is especially true in our case when there is no historical tract data available. Users should be aware of this.

To help data users assess our procedures, we publish all R and Python code used to construct this dataset and its validation (see below). Those wishing to run this code must register for a free account with the NHGIS (https://uma.pop.umn.edu/nhgis/user/new), request a free API key from the US Census Bureau (https://api.census.gov/data/key_signup.html), and obtain a standard Esri user license (https://pro.arcgis.com/en/pro-app/latest/get-started/about-licensing.htm) through their institution or via purchase. They will also need to install recent versions of R, ArcGIS Pro, and ArcGIS Desktop on their computers. We recommend that users run this code on an external hard drive with at least half a terabyte of memory, and we advise users that running the entire code will take several days. Further instructions are provided in our repository’s README files. These must be followed carefully for the code to run properly.

## Data Availability

All source code used in this analysis is available on Open Science Framework (OSF)^39^. This code was run using R 4.0.5, ArcGIS Pro 2.8 (with Python 3), and ArcGIS Desktop 10.7.
